# Sex differences in inflammatory parameters after shoulder arthroplasty and blood loss

**DOI:** 10.3389/fsurg.2024.1264443

**Published:** 2024-10-24

**Authors:** Stefan Hertling, Ekkehard Schleußner, Franziska Maria Loos, Niklas Eckhardt, Mario Kaiser, Isabel Graul

**Affiliations:** ^1^ Department of Obstetrics and Gynecology, University Hospital Jena, Jena, Germany; ^2^ Orthopedic Department, Campus Eisenberg, University Hospital Jena, Eisenberg, Germany; ^3^ Practice for Orthopedics and Shoulder Surgery Leipzig, Leipzig, Germany; ^4^ Institute for Diagnostic and Interventional Radiology, University Hospital Jena, Jena, Germany; ^5^ Module Integration Optics, Jenoptik Light and Optics Division, Jena, Germany; ^6^ Department of Trauma-, Hand- and Reconstructive Surgery, University of Jena, Jena, Germany

**Keywords:** shoulder arthroplasty, sex-specific, CRP, WBC, Hb

## Abstract

**Background:**

In many diseases, sex differences in diagnostics and therapy play role that is increasingly becoming recognized as important. C-reactive protein (CRP) and white blood cell (WBC) levels are determined as inflammatory markers to detect inflammation and even infection after total shoulder arthroplasty (TSA). The general course of white blood cell, CRP, and hemoglobin (Hb) levels after TSA is well known, but there is insufficient evidence of a possible association with sex. Therefore, we aimed to investigate whether there is an influence of sex on CRP, WBCs, and Hb after TSA in the first 10 days after surgery in a complication-free course in male and female patients and to re-evaluate the specific postoperative CRP, WBC, and Hb course with their maximums (minimum for Hb) and further course until the end of the inpatient period.

**Methods:**

We retrospectively studied patients treated with TSA, reverse shoulder arthroplasty (RSA), and prosthesis replacement between 2015 and 2021. Patients with active inflammation, rheumatoid arthritis, secondary osteoarthritis, active cancer, and documented postoperative complications were not included. CRP, WBC, and Hb levels before shoulder arthroplasty (SA) and up to 10 days after SA were recorded and analyzed for sex differences.

**Results:**

Data from a total of 316 patients (209 women and 107 men) were finally analyzed. There were no sex differences in the CRP and WBC values, but women had significantly lower preoperative Hb values, postoperative Hb values, and minimum Hb values. There were no significant differences in Hb, CRP, or WBC levels in the prosthesis exchange group.

**Conclusion:**

The progression of CRP and WBC levels showed no sex-specific significant differences after TSA within the first 7 postoperative days. The study confirmed a decreased Hb value for women at all stages of SA. Blood loss was significantly higher for RSA than for TSA for both men and women.

## Introduction

Surgical procedures, such as total shoulder arthroplasty (TSA) and inverse total shoulder arthroplasty (RSA), are used for various shoulder conditions, including end-stage shoulder arthropathy, cuff tear arthropathy, traumatic shoulder injuries, tumors, and failure of a previous arthroplasty. These procedures are associated with a postoperative risk of infection and blood loss (1). To monitor perioperative blood loss and inflammatory parameters after orthopedic surgery, particularly after major procedures such as joint replacement surgery, laboratory tests, especially blood sampling, are regularly performed (2). In this context, perioperative laboratory values and C-reactive protein (CRP) are established parameters. This acute-phase protein is involved in the early systemic inflammatory response (3). CRP is synthesized in hepatocytes—for example, in infectious processes and after tissue trauma or neoplasia (4). Local tissue damage leads to a cytokine cascade, with a rapid increase in serum CRP within 12–24 h at the end of the cascade (5, 6). Knowledge of the standard postoperative course of serum CRP after shoulder arthroplasty (SA) may be helpful in detecting unusual postoperative patterns. By determining the infection parameters, a normal course can be evaluated after certain operations. With the help of a normal course, abnormal postoperative inflammatory processes—for example, periprosthetic joint infections—can be detected and treated at an early stage (7, 8). Physiologic courses after various lower-limb surgical procedures have been described in the literature. After total knee arthroplasty (TKA) and total hip arthroplasty (THA), a peak in serum CRP levels was observed on the second or third day, which decreased in the following days (9). The difficulty in the increase in inflammatory scores after elective surgery such as shoulder arthroplasty is the ability to distinguish between the increase due to surgical trauma and that due to early-onset implant-associated infection. Larsson et al. showed a distinction could be made (10). In addition to iatrogenic influencing factors, external influencing factors and sex-specific influencing factors on the CRP course and further laboratory values were discussed. Sex differences seem to have a constant influence on the course of CRP and perioperative blood loss, but so far little research has been done, and at the same time, there is divergent discussion (11–13). In the field of shoulder surgery, the influence of sex differences on the perioperative course has been little investigated. Within shoulder surgery, shoulder arthroplasty is a growing field. Different types of prostheses and surgical procedures are used (14–17), some of which are already sex-specific. However, few data are available on the development of CRP and perioperative laboratory values after SA (18, 19). The aim of this study was to evaluate the influence of the choice of implanted shoulder prosthesis (anatomic or inverse) on postoperative laboratory values (CRP, leukocytes, and hemoglobin). Furthermore, it should be evaluated whether there is an influence of sex on the different shoulder prostheses. The results should help describe a sex-specific normal postoperative course of the standard laboratory parameters and assist in identifying postoperative complications in connection with an inflammatory reaction or blood loss that exceeds the normal level.

## Methods

### Data collection

This retrospective study included all patients who underwent elective SA in a single orthopedic center between 2015 and 2021. A total of 316 patients (209 men and 107 women) were included. Data regarding demographic characteristics, diagnosis, treatment, and laboratory parameters were retrieved from the hospital information system.

Exclusion criteria were defined as active infection (elevated CRP and joint puncture with bacterial detection or increase in granulocytes) of the shoulder or other infectious diseases (e.g., pneumonia or urinary tract infection) and neoplastic diseases.

If the initial CRP value was elevated, environmental diagnostics were performed to exclude infectious diseases. Thus, urine was tested for urinary tract infections, a chest X-ray was performed to exclude infiltrates of the lungs, and a puncture of the shoulder was performed to exclude bacterial colonization. If the diagnostic results were negative, surgical treatment was performed. These patients were included in the study. Osteoarthritis can also increase infection parameters and is a common underlying disease in cases of SA.

### Surgical treatment

All surgeries were performed by a surgeon experienced in shoulder arthroplasty.

The standard patient positioning is a modified beach chair position. SA was performed under general anesthesia. Antibiotic prophylaxis was administered as a single intravenous injection (cefuroxime 1.5 g, or clindamycin 600 mg in cases of penicillin allergy) through incision 30 min before the start of surgery. Standard postoperative antibiotic prophylaxis was not given.

The deltopectoral approach was used as standard access for all procedures. The cephalic vein was held away laterally and preserved when possible. Adequate hemostasis by coagulation was performed. Tenodesis of the long biceps tendon was performed. Tenotomy of the subscapularis tendon allowed visualization of the joint. In anatomic joint replacement (TSA), the subscapularis tendon was refixed as a standard procedure. In RSA, the subscapularis tendon was reattached when possible; tissue quality and soft tissue tension must be adequate for this purpose. Augmentation with polymethylmethacrylat (PMMA) bone cement was performed with a closed vacuum system (Copal R + G; Heraeus Medical GmbH, Wehrheim, Germany) according to the manufacturer's instructions. Thus, the risk of contamination should be reduced and homogeneous consistency achieved. Patients received a Redon drain (Megro/Ratiomed, Wesel, Germany) for at least 2 days postoperatively, depending on the amount of secretion. After SA, all patients were immobilized in an abduction pad for 4 weeks. After removal of the Redon drainage, the first clinical and radiographic follow-up examinations were carried out after the SA. A drainage system was performed as standard. Early functional physiotherapy was performed from the first postoperative day.

### Laboratory work

Laboratory tests included preoperative monitoring of the blood cell count, and serum CRP was collected for all patients.

Further blood samples were taken 4–5 times after the operation over the next 10 days. The inpatient treatment after TSA and RSA was approximately 10 days, and possibly longer after changing the prosthesis. The criterion for discharge from the hospital was the drop in the initially elevated parameters (CRP and leukocytes), and no signs that a local or systemic infection had occurred. Postoperative values for leukocytes, CRP, and Hb were evaluated in the study.

The measurements were used for statistical analysis to investigate a postoperative course and to identify a possible normal course of the leukocytes, CRP, and Hb in the serum after SA. In addition, the effects of sex and prosthesis-specific aspects (total shoulder arthroplasty, RSA, and prosthesis change) were analyzed.

### Statistical analysis

A statistical analysis of the data was performed using IBM SPSS Statistics, version 25 (IBM Corporation, Armonk, NY, USA).

The Mann–Whitney *U* test was used for non-parametric independent sampling of two groups, and the Kruskal–Wallis test was used to compare the distribution of leukocytes, CRP, and Hb in more than two groups (ANOVA). Otherwise, mean values are given with standard variation and compared using the Student's *t*-test for independent samples. The level of significance was set at the 5% level (*p* < 0.05). Charts were created with Microsoft Excel 2019 (Microsoft Corporation, Redmond, WA, USA). The patient data were displayed as a curve and a bar chart for postoperative CRP, WBC, and Hb values.

The study was approved by the university's ethics committee (University of Jena-2019-1370-BO).

## Results

### Demographics

A total of 316 patients were included; the mean age was 73 years (range 31–89 years). Of the patients, 209 were men (mean age 74 years; range 31–89 years) and 107 were women (mean age 68 years; age range 47–87 years).

A total of 89 patients underwent conventional SA, 192 patients underwent reverse shoulder arthroplasty, and 35 patients underwent a change of implanted shoulder arthroplasty. Demographic data are shown in Table 1.

**Table 1:** Demographic data of shoulder arthroplasty and prosthesis exchange subdivided by sex and age.

		*N*	Mean	Minimum	Maximum	Average	Standard deviation
RSA	Male	129	75	32	89	74.3	7.9
	Female	63	73	54	85	72.3	7.4
	Both sexes	192	75	32	89	73.6	7.7
SA	Male	56	67	31	86	65.6	13.1
	Female	33	60	47	80	60.1	9.4
	Both sexes	89	63	31	86	63.5	12.1
Exchange of SA	Male	24	72	50	82	69.7	8.6
	Female	11	68	54	84	69.4	8.5
	Both sexes	35	70	50	84	69.4	8.5
Total	Male	209	74	31	89	71.3	10.3
	Female	107	68	47	85	68.0	9.8
	Both sexes	316	73	31	89	70.2	10.2

### CRP

The baseline CRP, postoperative CRP, and maximum CRP values in the patients with RSA and TSA showed no significant difference between the sexes.

The baseline CRP values in the groups differed without significant differences between SA and RSA, but the exchange group had significantly higher preoperative CRP levels than the other groups. The postoperative maximum CRP levels were reached at a mean of 35.5 ± 30.9 mg/L. Subdivided within the groups, the highest postoperative CRP value was reached by conventional SA at a mean of 112.1 ± 70.7 mg/L, followed by RSA at 105.8 ± 62.2 mg/L and exchange of prosthesis at 82.1 ± 59.9 mg/L.

The maximum CRP level was reached on day 7.3 ± 1.5. There were no significant differences in the day the maximum CRP levels were reached in the subgroups. Although there was no significant difference in CRP level on the last-measured day (days 8–10), there was a significantly lower level of CRP in the group of exchanged prosthesis compared to SA and RSA.

Regarding only the women, there was no significant difference in preoperative CRP in the TSA, RSA, and exchange total endoprosthesis (TEP) groups, but a significant difference (*p* = 0.002) in the last-measured mean CRP in RSA at 39.1 ± 28.0 mg/dl compared to TSA at 23.5 ± 23.0 mg/dl. There was no difference in the maximum mean CRP achieved (RSA: 108.4 ± 71.7 mg/dl; TSA: 111.9 ± 65.8 mg/dl). In men, we did not find a difference in the last CRP level. However, in men, the baseline CRP level was significantly elevated in the prosthesis exchange group compared to the others, but not in the postoperative CRP levels.

**Figure 1:**
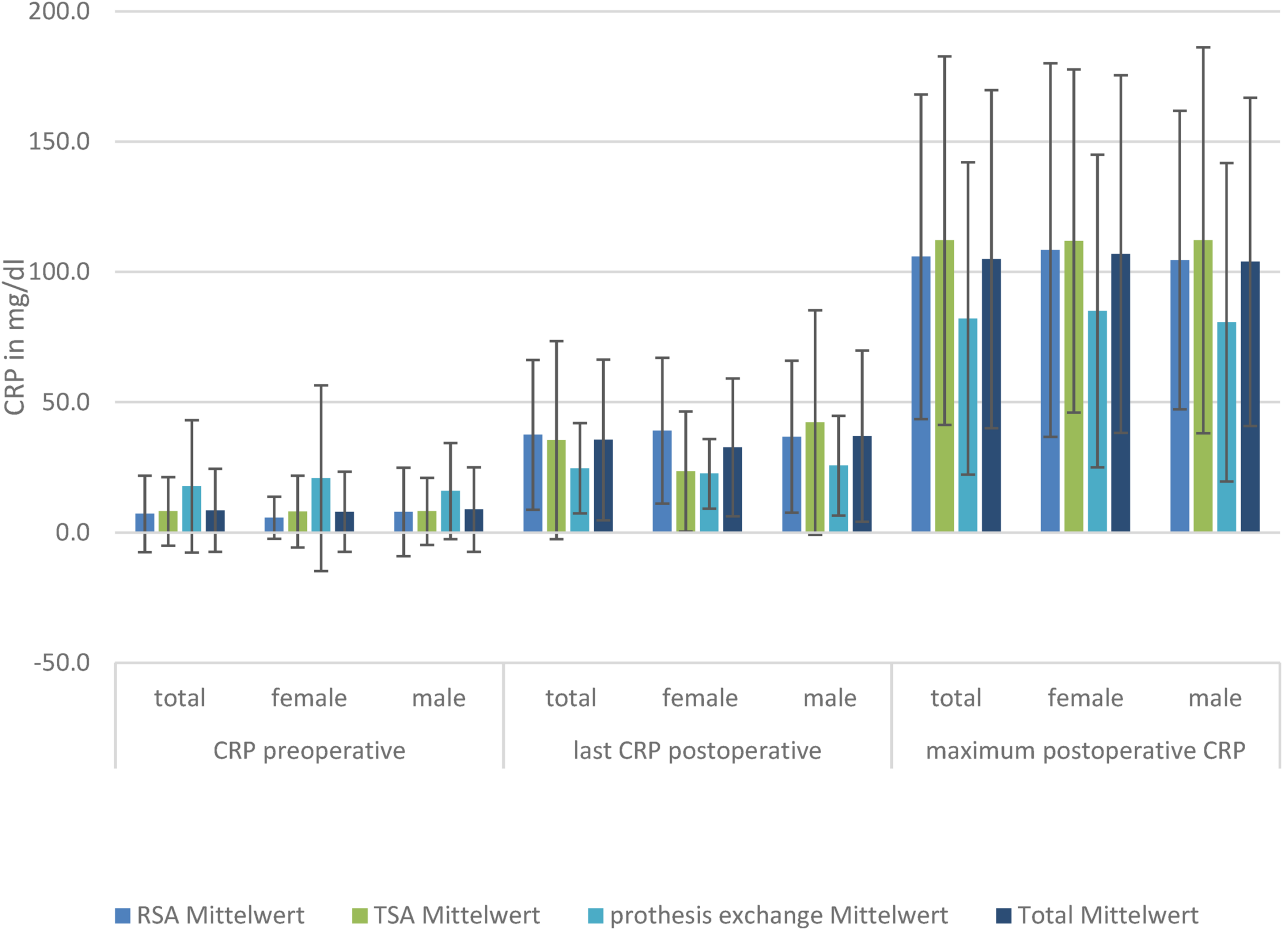
CRP levels in the three TSA, RSA, and prosthesis exchange groups: preoperative, postoperative, and maximum postoperative levels, divided by sex.

This significant CRP difference was not detectable in the group of male patients (Figure 1).

### White blood cell count

There was no significant difference in the preoperative, postoperative, or maximum WBC level in the TSA and RSA groups between the sexes.

**Figure 2:**
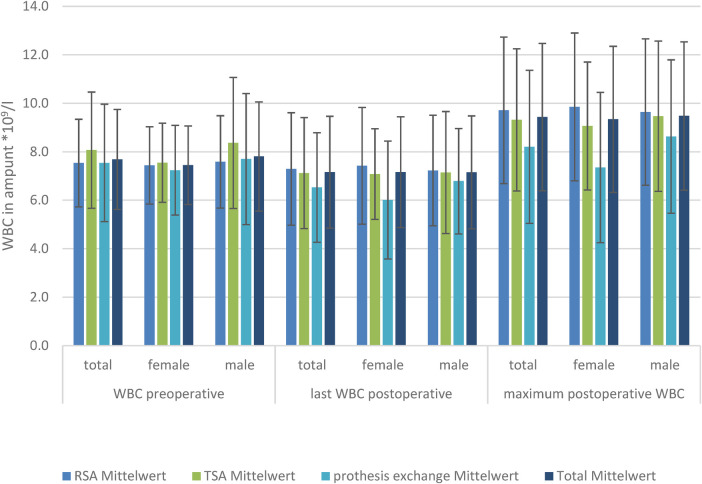
WBC levels in the three TSA, RSA, and prosthesis exchange groups: preoperative, postoperative, and maximum postoperative levels, divided by sex.

The baseline WBC level did not differ, nor did the postoperative CRP level at the last-measured day, and the highest measured WBC level differed in the TSA, RSA, and prosthesis exchange groups. Looking at the development of WBCs in the subgroups of men and women, there was also no significant difference in WBC level (Figure 2).

### Hemoglobin

There was a significant reduced hemoglobin and hematocrit in women in the TSA and RSA groups compared to men (*p* < 0.01) preoperatively, postoperatively, and in minimum Hb.

The baseline level of Hb is lowest in patients receiving prosthesis exchange (8.0 g/L), reaching a significant level compared to TSA (8.6 g/L; *p* = 0.01). Postoperative RSA (7.0 g/L) has a significantly lower Hb level than TSA (7.5 g/L) and prosthesis exchange (7.2 g/L). Considering the loss of blood measured by postoperative Hb compared with preoperative Hb, there was a significantly lower mean loss in the prosthesis exchange group (−0.30 ± 2.15 g/L; *p* < 0.01). However, there was no significant difference in mean blood loss between the TSA (−0.78 ± 1.94 g/L) and RSA (−1.33 ± 0.92 g/L) groups. When looking at the minimum Hb, there was a significantly lower mean Hb level reached in RSA (6.6 ± 1.1 g/L) compared to TSA (6.99 ± 1.24 g/L); the other groups showed no significant difference in minimum Hb. To show that the effect was not created by the massive dilution caused by fluid substitution, hematocrit was also analyzed and showed a significant decrease. Subdivided by sex, there is, in female patients, a significant difference in last-measured mean postoperative Hb (8.85 ± 0.97 g/L; *p* = 0.04) and blood loss for RSA (−1.6 ± 0.69 g/L) compared with TSA (−0.67 ± 2.33 g/L), but not for prosthesis exchange (−0.89 ± 1.64 g/L). However, the minimum mean Hb is significantly lower in prosthesis exchange (6.18 ± 1.99 g/L) compared with TSA (7.22 ± 1.58 g/L).

**Figure 3:**
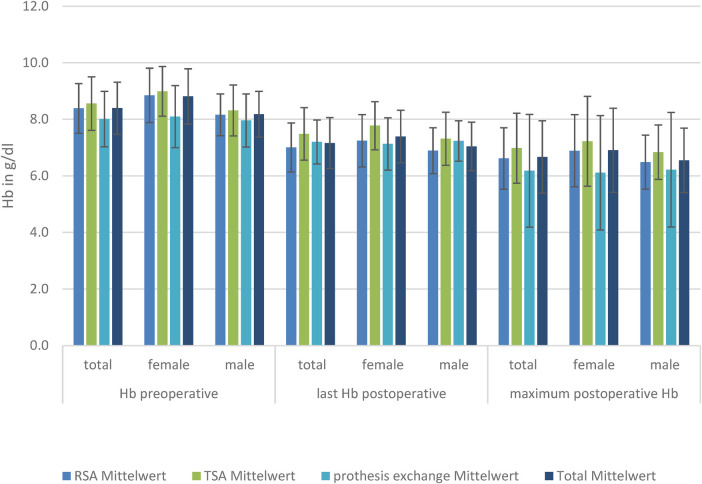
Hb levels in the three TSA, RSA, and prosthesis exchange groups: preoperative, postoperative, and maximum postoperative level, divided by sex.

Looking at the male patients, there was no significant difference in Hb level preoperatively, but the postoperative last-measured mean Hb level was significantly lower in RSA (6.89 ± 0.81 g/L) compared to TSA (7.31 ± 0.94 g/L; *p* = 0.05). Mean blood loss was significantly lower in the exchange group (0.0 ± 2.34 g/L) than in TSA (−0.85 ± 1.69 g/L) or RSA (−1.21 ± 0.99 g/L) (Figure 3).

## Discussion

The present study retrospectively examined the postoperative CRP, WBC, and Hb trends in the most common shoulder joint replacement surgeries of the anatomical and reverse shoulder prosthesis, as well as in the prosthesis changes, with special attention to sex-specific differences. The results of the present study can describe a normal postoperative course and assist in identifying postoperative complications in connection with an inflammatory reaction or blood loss that exceeds the normal level (2).

In general, existing epidemiological data show that sex differences play a crucial role in the immune response against viral, self, and tumor antigens, with women generally showing more robust innate and adaptive immune responses (20). Some studies have already shown that women have higher CRP levels than men (21, 22). However, whether this also leads to different CRP levels after postoperative stress is still unknown. In our study, we could not detect any differences in CRP or WBC values between men and women, either preoperatively or postoperatively. However, differences in CRP increases could be detected in the male and female groups.

The CRP level increases significantly more in women with RSA than with TSA; however, in men, the CRP level shows no difference. Higher operative stress leads to higher CRP secretion in women. Comparing only CRP values preoperatively and postoperatively after 8–10 days and the highest CRP value achieved, men and women did not differ. This means that it is not the absolute CRP value that diverges between men and women, but that there is a higher increase in CRP among women in the RSA group. Greater trauma, as can be expected for inverse SA compared with conventional SA, does not necessarily lead to higher CRP values. Otherwise, the group undergoing prosthesis replacements, with significantly increased trauma, would also have higher CRP values. However, this statement can only be interpreted in this way to a limited extent, since antibiotics (or at least cements containing antibiotics) are frequently used in denture changes, even without evidence of germs. This could have an influence here, which limits the results of a CRP increase depending on the trauma, as Tursich et al. showed in their meta-analysis of 13,374 patients that CRP levels increase after trauma (23), though they did not conduct a sex-specific analysis.

However, we demonstrated a significant difference in the increase in WBCs preoperatively from the last-measured value: WBC levels remained highest in the RSA group until days 8–10.

Regarding the hemoglobin value, a lower value was already shown preoperatively in patients undergoing exchange surgery. However, patients who undergo replacement surgery have usually had multiple prior shoulder surgeries for a variety of reasons and have often had long courses of antibiotic therapy. In the present study, the reasons for the change operations were not considered individually, and the group was only used for comparison with the primary shoulder operations performed here. Interestingly, patients after RSA showed significantly lower Hb values on day 10 than patients with conventional SA as well as a lower minimum Hb postoperatively. Since the comparison of hematocrit values cannot confirm a dilution effect, a higher blood loss of RSA compared to SA can be assumed here. Kim et al. showed that the mean overall blood loss in RSA was 846.6 ± 527.6 ml, which included 346 ± 231.2 ml of intraoperative blood loss and 500.3 ± 196.4 ml of postoperative blood loss (24). A total of 278 patients analyzed by Malcherczyk et al. showed a mean total blood loss of 392.7 ml in reverse SA, 394.6 ml in anatomical SA, and 298.3 ml in stemless SA (25). It can be speculated that the overall higher infection levels after RSA may be related to the more invasive surgical technique due to larger implant sizes. In their study, Niskanen et al. described a characteristic serological CRP pattern in patients after elective TKA and THA, with a peak on the second and third postoperative day (26), as Klingebiel et al. did for SA (27). Our SA data showed a similar pattern, with a peak on day 7.

An interesting fact is that in our study, mostly RSA procedures were implanted and conventional SA was used significantly less often; this distribution is not evident from other studies. Why RSA procedures were implanted more frequently in this study cannot be adequately explained retrospectively. An evaluation of the underlying problems and of the intact rotator cuffs leading to these procedures was not performed.

A study by Windisch et al. (28) evaluated sex differences in postoperative trends in more than 1,000 primary TKA procedures and found, similar to the results of Rohe et al. (29), that male patients had significantly higher CRP peaks after TKA or THA. The sex differences were explained in the studies by larger wound areas and more bone resection in male patients due to their larger body sizes. Male patients showed significantly higher CRP peaks, in contrast to our present findings. These findings are not supported by the present data that consider SA. Our findings are in line with the findings of Klingebiel et al. who, in 2020, also examined SA (27).

### Strengths and limitations

Because the operations examined were all performed in a single center, it can be assumed that the surgical techniques for the individual modalities did not differ significantly. A major strength of the study is the high proportion of RSA, which is usually much lower in the evaluations of similar studies. The follow-up time of 10 days is enough for a normal postoperative follow-up of the blood parameters, but not long enough to talk about postoperative inflammation. The fact that the influence of different surgeons and operation time were not considered is a limitation to the study. In addition, influencing parameters, such as other diseases (e.g., diabetes) or body mass index, were not considered.

## Conclusion

There is a serum CRP trend after SA, with a peak on the seventh day and a decline by the tenth day. RSA and conventional SA showed no differences in the trend of inflammation levels (leukocytes and CRP). Inflammation levels after SA were not dependent on sex. The presented results may contribute to a better understanding of the postoperative course after shoulder arthroplasty. Hemoglobin loss, and thus blood loss, in RSA is statistically higher than in conventional SA. The results of this retrospective study may help improve the diagnosis and treatment of infections and blood loss after shoulder arthroplasty in a sex-specific manner. Further studies with prospective designs and longer follow-ups are needed for this purpose.

## Data Availability

The raw data supporting the conclusions of this article will be made available by the authors, without undue reservation.
